# Network Meta-Analysis of Different Clinical Commonly Used Drugs for the Treatment of Hypertrophic Scar and Keloid

**DOI:** 10.3389/fmed.2021.691628

**Published:** 2021-09-09

**Authors:** Sha Yang, Yujia J. Luo, Cong Luo

**Affiliations:** ^1^Department of Orthopaedics, Children's Hospital of Chongqing Medical University, Chongqing, China; ^2^Ministry of Education Key Laboratory of Child Development and Disorders, National Clinical Research Center for Child Health and Disorders, China International Science and Technology Cooperation Base of Child Development and Critical Disorders, Chongqing Key Laboratory of Pediatrics, Chongqing Engineering Research Center of Stem Cell Therapy, Children's Hospital of Chongqing Medical University, Chongqing, China; ^3^Department of Neurosurgeons, Children's Hospital of Chongqing Medical University, Chongqing, China

**Keywords:** network meta-analysis, efficacy, safety, drugs, hypertrophic scar, keloid

## Abstract

**Background:** There is no uniform treatment for pathological scars, including keloids and hypertrophic scars, in clinic currently. Previously, multiple randomized controlled trials have examined the clinical efficacy of different treatments. Nonetheless, the results are inconsistent, and many treatments have not been directly compared. This makes it difficult to conclude which approach is more favorable, in terms of efficacy and safety, for the treatment of pathological scarring. This study aimed at evaluating the efficacy of different injection and topical treatment strategies for hypertrophic scar and keloid.

**Methods:** Relevant literature from PubMed, Medline, Embase, Scopus, the Cochrane Central Register of Controlled Trials (CCRCT), and WHO International Clinical Trials Registry Platform (WHO-ICTRP) were searched, from database inception through November 2020. Randomized clinical trials evaluating different treatment strategies of pathological scars, including triamcinolone acetonide (TAC), verapamil (VER), 5-fluorouracil (5-FU), botulinum toxin A (BTA), bleomycin (BLM), and silicone gels were included in the study.

**Results:** The network meta-analysis included a total of 2,009 patients from 29 studies. A network meta-analysis of injection and topical treatment strategies showed that the efficacy of TAC combined with BTA was best in the treatment of pathological scars. Combination therapies of TAC with 5-FU and TAC with BTA significantly improved the clinical efficiency. However, there was no statistically significant difference between other treatment strategies. The order of efficacy predicted by the surface under the cumulative ranking (SUCRA) curve was as follows: TAC+BTA (82.2%) > TAC+5-FU (69.8%) > BTA (67.3%) > 5-FU+silicone (59.4%) > TAC+silicone (58.3%) > 5-FU (49.8%) > BLM (42.0%) > TAC (26.7%) > VER (26.2%) > silicone (18.3%). There was no publication bias revealed based on the funnel diagram.

**Conclusion:** This study recommends intralesional injection of TAC-BTA and TAC-5-FU combined therapies. But for patients who cannot tolerate the side effects, the use of silicone gels in combination with TAC is recommended. However, these conclusions need to be further confirmed by more randomized controlled trials.

## Introduction

Pathological scars, including hypertrophic scars and keloids, are mostly caused by the formation of a large extracellular matrix and proliferation of fibroblasts. The formation is characterized by uncontrolled deposition of collagen resulting from destruction of the balance between the catabolic and anabolic effects of collagen during wound healing (1). Patients with keloids and hypertrophic scars may present with symptoms such as pain, erythema, itching, and the persistent growth of lumps. These may lead to malformations in appearance and function as well as cause physical and psychological pain to the patient. Severe symptoms can affect the self-confidence of patients, leading to inferiority complex and seriously affecting the quality of life and mental health (2). The mechanism of pathological scar formation is not fully understood (3, 4). Some studies have suggested that the formation of scar may involve a variety of cells like fibroblast, myofibroblast, and mastocyte (5, 6), cytokines like transforming growth factor-β (TGF-β2), and tumor necrosis factor-α (7–9), extracellular matrix (deposition of collagen and glycolaminoglycan) (10), and spatial structure of tissue (excessive angiogenesis and repair of the spatial network between cells) (11). Demographic characteristics, such as race, sex, and age, as well as external factors like type of injury, also may play a significant role in scar formation (12, 13). Darker skin phenotypes, namely, Fitzpatrick's phototype classification (1975) and skin types IV and V (moderately pigmented), are prone to pathological scars after injury. In addition, women and teenagers are more prone to scars than men and adults, respectively. Elsewhere, keloids can be seen with chronic inflammatory skin diseases such as hidradenitis suppurativa and have been reported to appear spontaneously without trauma, mostly with syndromes (14–16).

There are many treatments available for keloid and hypertrophic scars, including topical therapy, drug injections, pressure therapy, laser therapy, surgery, and cryotherapy (17). Silicone gels are commonly used for topical treatment. Specifically, they are considered as first-line agents for the treatment of mild hypertrophic scars (14, 15, 18, 19). Silicone dressing can be offered to patients with predisposition to develop keloid or hypertrophic scar after future any surgical intervention. Silicone dressing is hypothesized to act by hydrating the wound, inhibiting collagen deposition, and downregulating TGF-β2 (20). Hydrated and occluded environment impairs capillary activity and the continuation of subsequent pathological regeneration signals (21). This affects fibroblast regulation and reduces synthesis of collagen (22). Hydration stabilizes mastocytes, thereby causing suppressive effects on inflammation (23). Silicone gel therapy is offered to patients as a prophylaxis and as a non-invasive treatment following excessive scarring. It is worthy to note that previous studies reported conflicting findings on silicone gels' efficacy (20). Drug injection commonly used includes triamcinolone acetonide (TAC), 5-fluorouracil (5-FU), botulinum toxin A (BTA), bleomycin (BLM), and verapamil (VER). TAC, the most used corticosteroid for the treatment of keloids and hypertrophic scars (24, 25), acts by anti-inflammatory and antimitotic mechanisms. It inhibits the growth of fibroblasts and reduces endothelial budding and synthesis of procollagen and glycosaminoglycan. Also, it enhances the degeneration of collagen and fibroblasts (26) and triggers a significant decrease in VEGF, α-1-antitrypsin, and α-2-macroglobulin levels (27–30).

The 5-FU is a pyrimidine analog that inhibits thymidine synthase, which inhibits nucleic acid synthesis and cell proliferation. *In vivo* and *in vitro* experiments have shown that it inhibits fibroblast proliferation, angiogenesis, and TGF-β-induced collagen type I expression, while increasing fibroblast apoptosis (31). BTA, isolated from *Clostridium botulinum* (32), is a potent neurotoxin that can block neuromuscular conduction. It can make fibroblasts stationary in G0 and G1 phases of the cell cycle (33) and non-proliferative state. This is realized by reducing the tension at the edge of the healing wound (34), which reduces the expression of TGF-β1, thereby inhibiting scar formation (35, 36). BLM, derived from *Streptomyces verticillus*, is cytotoxic to keratinocytes and eccrine epithelial cells (37, 38). It is an antitumor, antiviral, and antimicrobial agent that inhibits DNA, RNA, and protein synthesis. It also reduces the level of lysine oxidase, a cross-linked enzyme involved in collagen maturation (39). VER is a calcium channel blocker that has been shown to increase the synthesis of pro-collagenase in keloid, hypertrophic scars, and normal cultured fibroblasts. This leads to actin filamolymerization, cell conformational changes, and apoptosis and ultimately reduced fibrous tissue production (40, 41). It may also inhibit the frequently elevated cytokines in keloids, such as IL-6 VEGF and TGF-β1 (42).

The above treatment methods can be used as monotherapy or combination therapy. Studies have shown that each method has different degrees of efficacy (43), and each method has its corresponding side effects. However, literature regarding these methods in management of hypertrophic scars and keloids is often limited. They include small patient numbers, lack blinding and controls, and assume retrospective design and insufficient follow-up time. Also, some include both keloids and hypertrophic scars within the same treatment group. Keeping these confounding factors in mind, these studies have shown that the aforementioned methods can be efficacious in preventing the development of as well as reducing existing hypertrophic scars and keloids.

When comparing the advantages and disadvantages of three or more interventions, traditional meta-analysis can no longer provide a definite answer. Unlike the traditional meta-analysis, network meta-analysis (NMA) has the advantage that it can use direct and indirect comparison methods to rank the efficacy of different interventions and provide an overview of the optimal plan (44). There have been previous mesh meta-analyses on similar topics (45, 46), but the interventions evaluated are different from ours, and new interventions are constantly being developed for the prevention and treatment of scarring. In this study, an NMA was used to evaluate the efficacy and tolerability of TAC, 5-FU, BLM, silicone gel, BTA, and VER. Furthermore, their two-drug combination therapy, including effective rate and adverse reaction rate and recurrence rate, was explored. The findings of this study provide a clinical scientific and reliable summary that can help guide treatment decisions.

## Materials and Methods

### Search Strategy

This study conforms to the Preferred Reporting Items for Systematic Reviews and Meta-Analyses (PRISMA) guidelines (46). The existing studies from EMBASE, PubMed Medline, Scopus, the Cochrane Central Register of Controlled Trials (CCRCT), and WHO International Clinical Trials Registry Platform (WHO-ICTRP) electronic databases were searched in accordance with the Cochrane Collaboration criterion. Randomized controlled trials (RCTs) investigating the safety and efficacy of all current therapies used for the management of hypertrophic scars and keloids, from database inception through November 2020, were included in the study. Besides, the reference lists for the identified RCT were also hand-searched for possible relevant articles to avoid relevant information being missing. The search was only limited to human studies, and no language restrictions were posted on the setting. The document retrieval adopted the combination of subject word and random word. The search query was formulated based on the following keywords: “scar” or “hypertrophic scars” or “keloids” or their synonyms and “scar management” or “Triamcinolone Acetonide” or “Verapamil” or “5-fluorouracil” or “Botulinum Toxins Type A” or “Bleomycin” or “Silicone” or their synonyms.”

### Inclusion and Exclusion Criteria of Studies

#### Inclusion Criteria

(1) RCT, no language limitation; (2) any patient with hypertrophic scar or keloid, regardless of age, regardless of whether the diagnosis of the scars was clinical or histopathological; (3) TAC or VER or 5-FU or BTA or BLM or silicone, whether it is the monotherapy of the above therapies or the combination of the two therapies; and (4) outcome indicators: must include effective rate, including but not limited to adverse reaction rate and recurrence rate. The effective rate of each therapeutic strategy was calculated using the following formula: [*n* (effective events)/*n* (total events)]. *Exclusion criteria*: (1) case control studies, case reports, abstracts from conference proceedings, and non-human studies, among others; (2) research on special populations, such as pregnant women with immunodeficiency; (3) study on non-above five interventions; (4) lack of relevant outcome data; and (5) <10 people per group.

### Data Extraction and Quality Assessment

Two reviewers (YS and LYJ) extracted data independently using a predefined data extraction form. After the duplicates were eliminated, qualified studies were preliminarily screened, full text was downloaded by browsing the titles and abstracts, and finally, the included studies were determined. Disagreements were resolved by discussion or consensus with a third reviewer (LC). The data extracted included the first author; country, study characteristics (i.e., year and duration); participant characteristics (i.e., mean age, proportion of male and sample size); of the experimental and control group treatments; and measured outcomes. For studies with insufficient information, the reviewers contacted the primary authors, when possible, to acquire and verify the data.

### Statistical Analysis

Data for the included literature were analyzed using R software and Stata software. The heterogeneity was evaluated using the *I*^2^ test, with *I*^2^ < 50% scoring for low heterogeneity, while *I*^2^ >50% scoring for significant heterogeneity. The risk ratio (RR) was used as an effect statistical index for the effective rate; adverse reaction rate, recurrence rate, and its 95% confidence interval (CI) were calculated. Four Markov chains were used for the initial value setting model. The number of iterations for the first update was set as 50,000; and the number of iterations for the further update was set as 100,000. The first 50,000 annealing times were used to eliminate the influence of the initial value, and the sampling started from 50,001. The random-effects model was used within Bayesian NMA. A random-effects model with small deviance information criterion (DIC) was selected in this study. The smaller the DIC value, the better the model fitting effect (47). Convergence of iteration was evaluated using the Brooks–Gelman–Rubin method. In the case of a closed loop, the consistency between direct and indirect comparisons was judged based on node analysis. A *p*-value < 0.05 scored positive for inconsistency; thus, the inconsistency model was used for analysis. Local inconsistency was tested using the node-split Model, and a *p*-value >0.05 indicated that the heterogeneity of the included studies was small, so the consistency model was used for analysis. The potential scale reduction factor (PSRF) reflects convergence. When PSRF is close to 1 or equal to 1, it indicates that good convergence efficiency has been achieved, and the reliability of the results obtained using consistency model analysis is high (48). A scatter plot was drawn according to surface under the cumulative ranking of the efficacy and tolerance of each therapeutic measure. The bias risk of the included literature was determined using Reviewer Manager (RevMan) 5.3 software. Publication bias was evaluated with a funnel plot (49). A *p*-value < 0.05 was regarded as statistically significant.

## Results

### Literature Search and Characteristics of Included Studies

**Figure 1:**
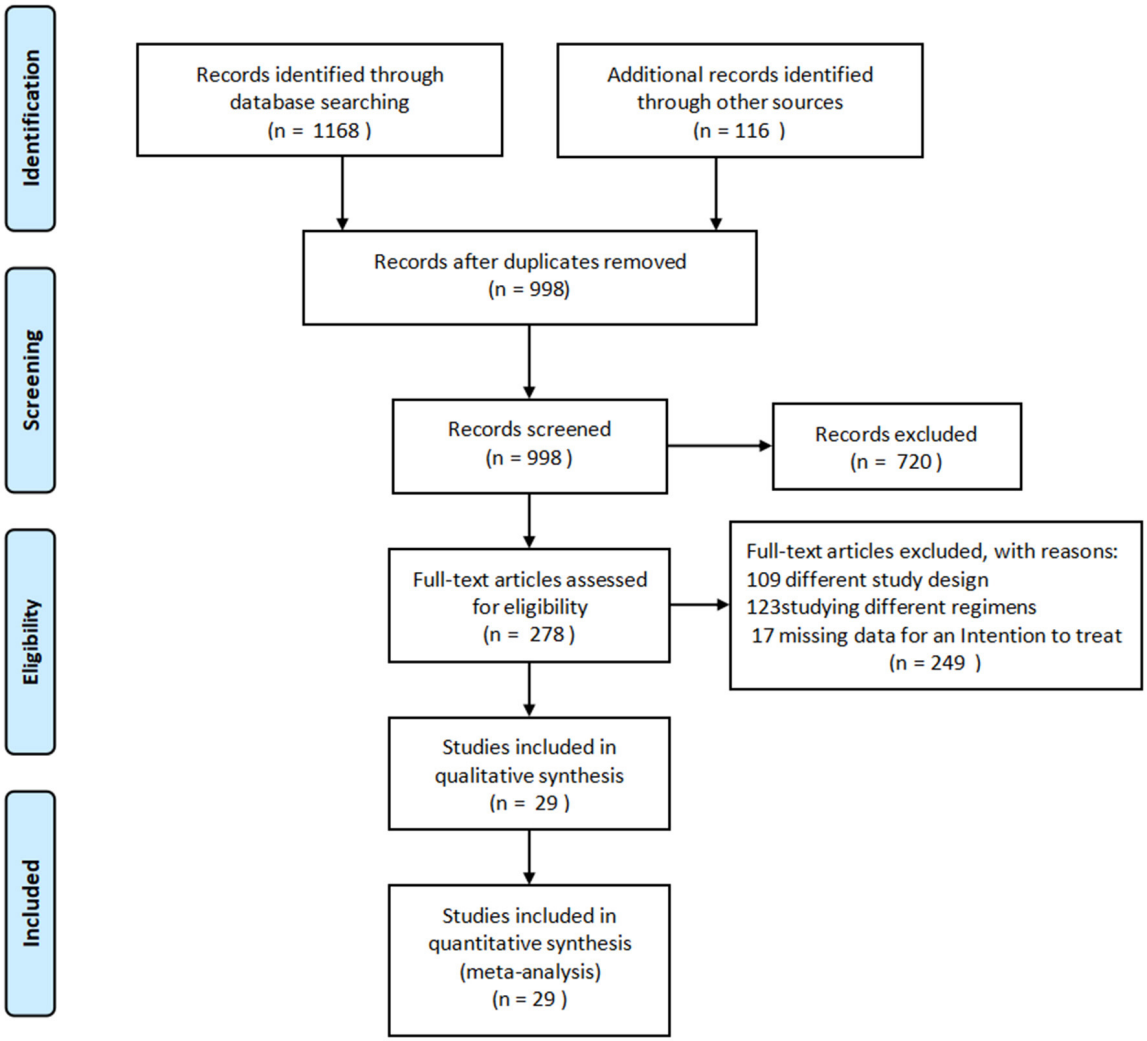
PRISMA flow diagram of the study selection process. PRISMA, Preferred Reporting Items for Systematic Reviews and Meta-Analyses.

**Figure 2:**
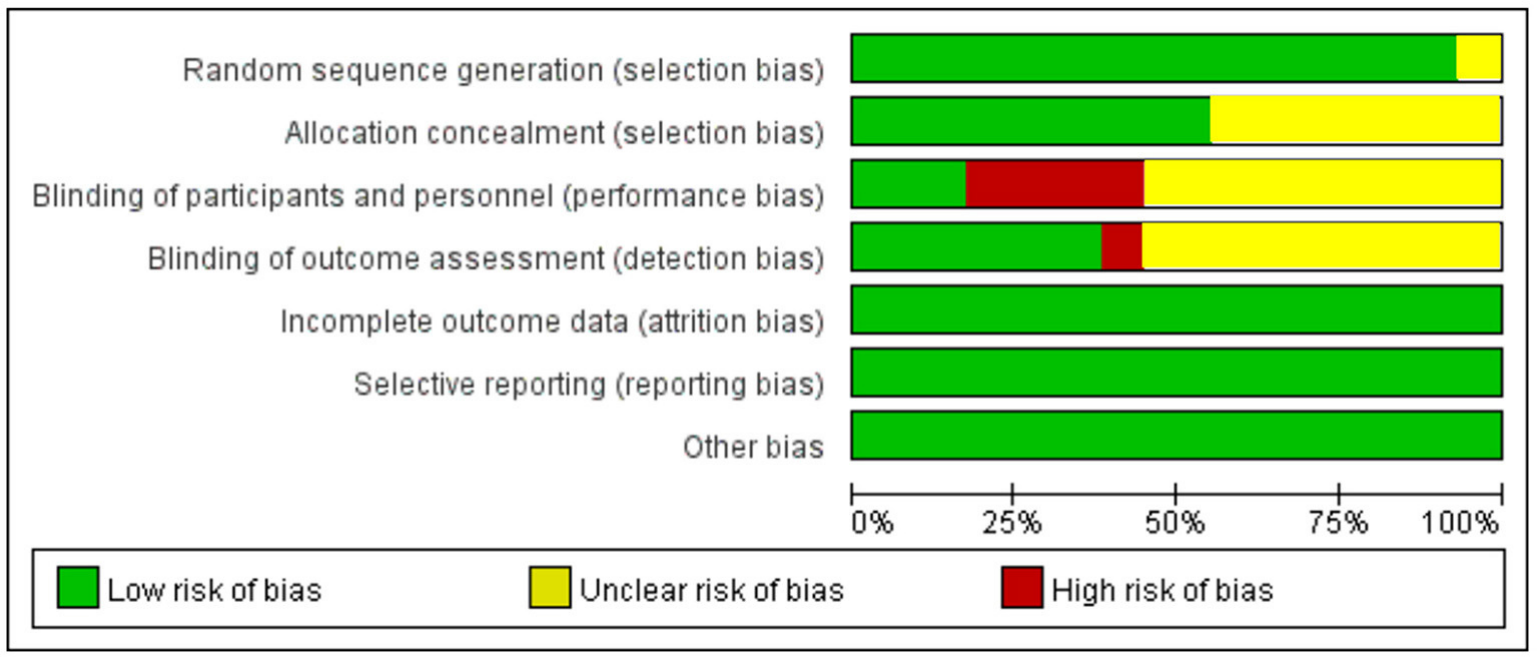
Summary of risk of bias assessment.

A detailed overview of the selection process is shown in Figure 1. A total of 29 studies, including 2,009 patients, were enrolled in the meta-analysis. The characteristics and methodology assessment of individual studies included in the meta-analysis are described in Table 1. The risk of bias assessment is shown in Figure 2.

**Table 1:** Characteristics of the 29 included studies.

**No**.	**References**	**Country**	**Participants (male/female)**	**Mean age (range) year**	**follow-up**	**Outcomes**	**Interventions**
1	Hietanen et al. (50)	Finland	5-FU 25 (15/10) TAC 25 (11/14)	46.96 (21–81) 41(18–71)	6m	Effective rate	5-FU vs. TAC
2	Manuskiatti and Fitzpatrick (51)	Thailand	5-FU+TAC 10 (4/6) 5-FU 10 (4/6) TAC 10 (4/6)	(25–74)	32w	Effective rate	5-FU+TAC vs. 5-FU vs. TAC
3	Saha and Mukhopadhyay (52)	India	5-FU 20 TAC 24	34.7 (16–66) 32.96 (19–60)	12m	Effective rate	5-FU vs. TAC
4	Sadeghinia and Sadeghinia (53)	Iran	5-FU 20 TAC 20	43.36 45	44w	Effective rate? Adverse effects rate	5-FU vs. TACv
5	Zhu et al. (54)	China	5-FU+TAC 46 (25/21) TAC 46 (26/20)	30.25 (15–51) 42.37 (16–68)	12w	Effective rate? Adverse effects rate	5-FU+TAC vs. TAC
6	Deng (55)	China	5-FU+TAC 48 (26/22) TAC 48 (25/23)	33.7 (17–56) 32.5 (15–52)	12m	Effective rate, Adverse effects rate	5-FU+TAC vs. TAC
7	Darougheh et al. (56)	Iran	5-FU+TAC 20 (15/25) TAC 20	25.2 23.4	12w	Effective rate, Adverse effects rate	5-FU+TAC vs. TAC
8	Khalid et al. (57)	Pakistan	5-FU+TAC 60(26/34) TAC 60 (25/35)	31.22 27.67	12w	Effective rate, Adverse effects rate, Recurrence rate	5-FU+TAC vs. TAC
9	Asilian et al. (58)	Iran	5-FU+TAC 20 (8/12) TAC 20 (7/13)	25.3 23.4	12w	Effective rate, Adverse effects rate, Recurrence rate	5-FU+TAC vs. TAC
10	Khan et al. (59)	Pakistan	5-FU+TAC 75 (65/85) TAC 75	28.96 29.93	12w	Effective rate, Adverse effects rate	5-FU+TAC vs. TAC
11	Shilin et al. (60)	China	5-FU+TAC 60 (28/32) TAC 60 (29/31)	26.82 (14–54) 31.25 (15–58)	24m	Effective rate, Adverse effects rate, Recurrence rate	5-FU+TAC vs. TAC
12	Khan et al. (61)	Pakistan	BLM 82 (31/51) TAC 82 (33/49)	32 33	24w	Effective rate	BLM vs. TAC
13	Payapvipapong et al. (37)	Thailand	BLM 14 (9/5) TAC 12 (7/5)	29.8 38.4	12w	Effective rate, Adverse effects rate	BLM vs. TAC
14	Hatamipour et al. (62)	Iran	Silicone+5-FU 25 (20/30) Silicone 25	(22–45)	12m	Effective rate, Adverse effects rate, Recurrence rate	silicone+5-FU vs. silicone
15	Xiaohui et al. (63)	China	Silicone+TAC 30(12/18) Silicone 30(10/20)	41.34 39.86	6m	Effective rate, Adverse effects rate, Recurrence rate	Silicone+TAC vs. Silicone
16	Zhang et al. (64)	China	TAC 35 (26/44) Silicone 35	(19–47)	3m	Effective rate, Adverse effects rate	TAC vs. Silicone
17	Gamil et al. (65)	Egypt	BTA+TAC 24 (16/8) BTA 26 (18/8) TAC 26 (18/8)	27.8 (20–35) 28.4 (19–43) 28.4 (19–43)	12w	Effective rate Adverse effects rate, Recurrence rate	BTA+TAC vs. BTA vs. TAC
18	Cheng (66)	China	BTA+TAC 23 (21/25) TAC 23	27.5 (18–46)	6m	Effective rate	BTA+TAC vs. TAC
19	Li et al. (67)	China	BTA+TAC 37 (17/20) TAC 37 (16/21)	25.2 (18–35) 26.04 (20–33)	6m	Effective rate	BTA+TAC vs. TAC
20	Yuqin et al. (68)	China	BTA 41 (26/15) TAC 41 (25/16)	31.69 (22–41) 31.12 (19–56)	6m	Effective rate, Adverse effects rate, Recurrence rate	BTA vs. TAC
21	Bin et al. (69)	China	BTA 40 (51/39) TAC 40	29.3(18-53)	6m	Effective rate, Adverse effects rate, Recurrence rate	BTA vs. TAC
22	Nai-Kang et al. (70)	China	BTA 40 (16/24) TAC 40 (13/27)	30.78 (18–50) 31.45 (18–49)	4m	Effective rate, Adverse effects rate	BTA vs. TAC
23	Shaarawy et al. (71)	Egypt	BTA 12 (0/12) TAC 12 (0/12)	29.29 (10–53)	7m	Effective rate, Adverse effects rate	BTA vs. TAC
24	Aggarwal et al. (72)	India	VER 15 TAC 16	–	24w	Effective rate, Adverse effects rate	VER vs. TAC
25	Abedini et al. (40)	Iran	VER 50 TAC 50	(18–65)	3m	Effective rate, Adverse effects rate, Recurrence rate	VER vs. TAC
26	Margaret Shanthi et al. (73)	India	VER 27 TAC 27	26 20	12m	Effective rate	VER vs. TAC
27	Chunan et al. (74)	China	VER 17 TAC 17	–	13m	Effective rate, Adverse effects rate, Recurrence rate	VER vs. TAC
28	Ahuja et al. (74)	India	VER 25 TAC 21	–	24w	Effective rate	VER vs. TAC
29	Tao et al. (75)	China	VER 45 (19/26) TAC 45 (21/24)	27.89 (12–43) 28.47 (13–45)	NA	Effective rate, Adverse effects rate	VER vs. TAC

*m: months, w: weeks*.

### Effective Rate

**Figure 3:**
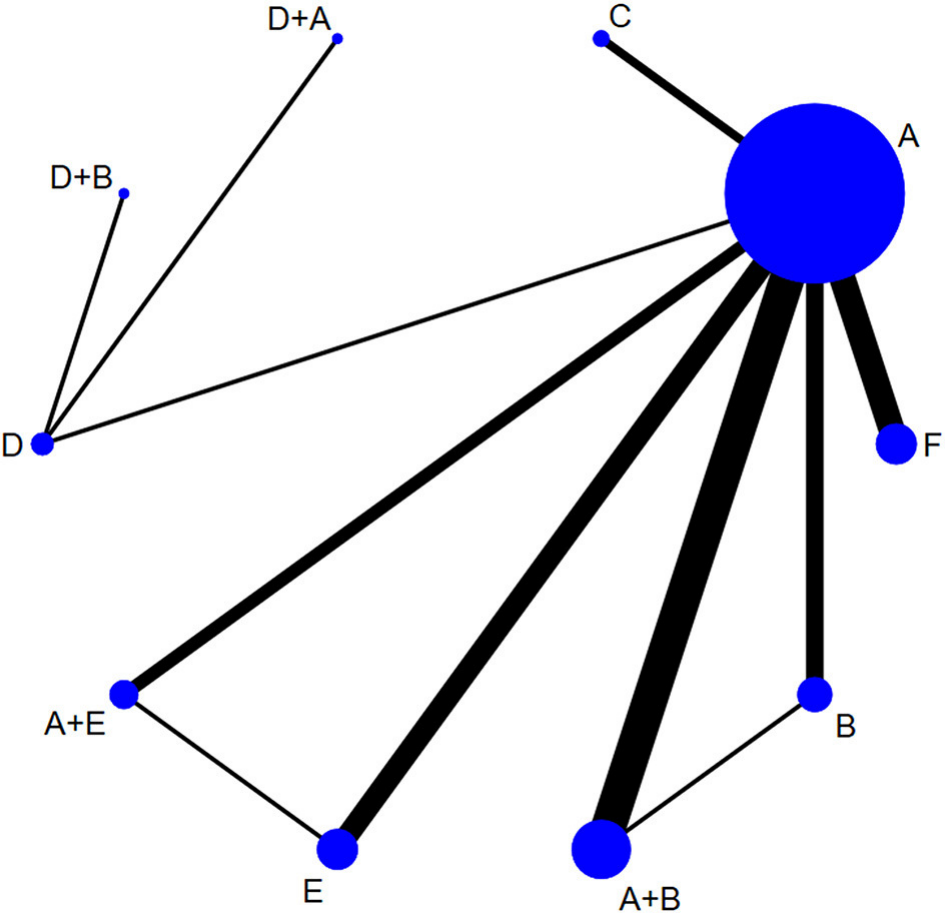
Network diagram of effective rate. A, TAC; B, 5-FU; C, BLM; D, silicone; E, BTA; F, VER; A+B, TAC+5-FU; D+A, silicone+TAC; D+B, silicone+5-FU; A+E, TAC+BTA. TAC, triamcinolone acetonide; 5-FU, 5-fluorouracil; BLM, bleomycin; VER, verapamil.

Effective rates were reported in 29 studies (37, 40, 50–76), including six monotherapy measures and four combination measures. The former comprised A, TAC; B, 5-FU; C, BLM; D, silicone gels; E, BTA; and F, VER. The latter comprised A+B, TAC combined with 5-FU; D+A, silicone gels combined with TAC; D+B, silicone gels combined with 5-FU; and A+E, TAC combined with BTA. The network diagram of effective rate is shown in Figure 3. The 10 treatment measures formed 45 pair comparisons. In terms of treatment effectiveness, there were 11 direct comparisons among 29 studies, and the rest were indirect comparisons.

#### Detection of Inconsistency

Eleven direct comparisons constituted two triangular closed loops. The Z-test results showed that the lower limits of 95% CI were 0. This implies that each closed loop was consistent, as shown in Table 2. The results of the node-splitting model showed consistency in the direct and indirect comparison results all at *p* > 0.05 (Table 3).

**Table 2:** Inconsistency detection of closed loop for effective rate.

**Loop**	***p*-value**	**95% CI**
TAC−5-FU–TAC+5-FU	0.194	(0.00, 6.06)
TAC–TAC+BTA—BTA	0.221	(0.00, 3.56)

**Table 3:** Results of node splitting model for effective rate.

**Side**	**A B**	**A C**	**A D**	**A F**	**A I**	**B D**	**C I**	**F G**	**F H**
*p*-value	0.593	0.221	0.171	1.000	0.768	0.259	0.544	1.000	1.000

*A, TAC; B, TAC+5-FU; C, TAC+BTA; D, 5-FU; F, Silicone; G, TAC+Silicone; H, Silicone+5-FU; I, BTA*.

#### Results of Network Meta-Analysis and Publication Bias

**Figure 4:**
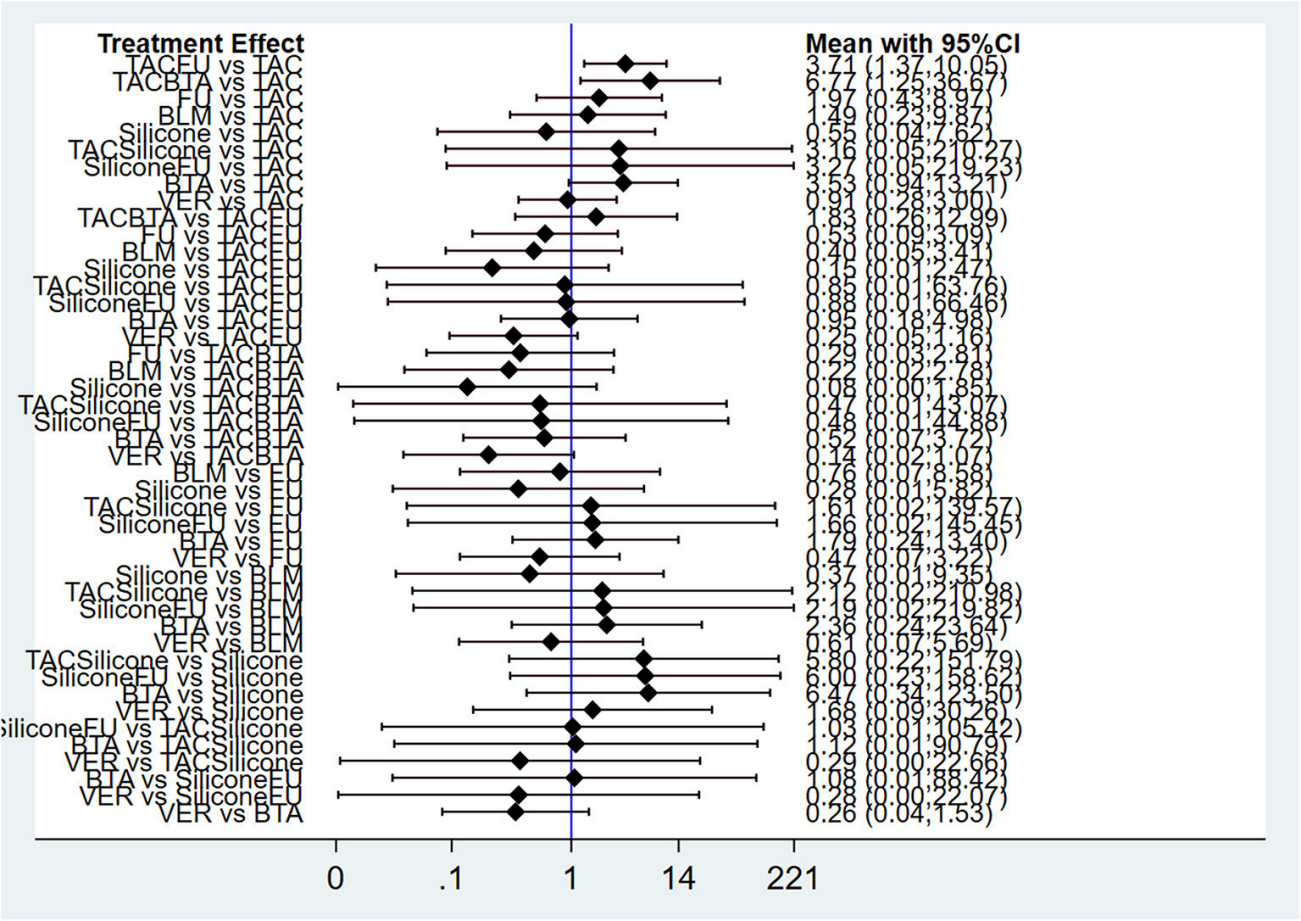
Forest plot of efficacy rate for pairwise treatment comparison. FU, 5-FU; TACFU, TAC+5-FU; TACBTA, TAC+BTA; TACSilicone, TAC+silicone; SiliconeFU, silicone+5-FU. TAC, triamcinolone acetonide; 5-FU, 5-fluorouracil.

**Figure 5:**
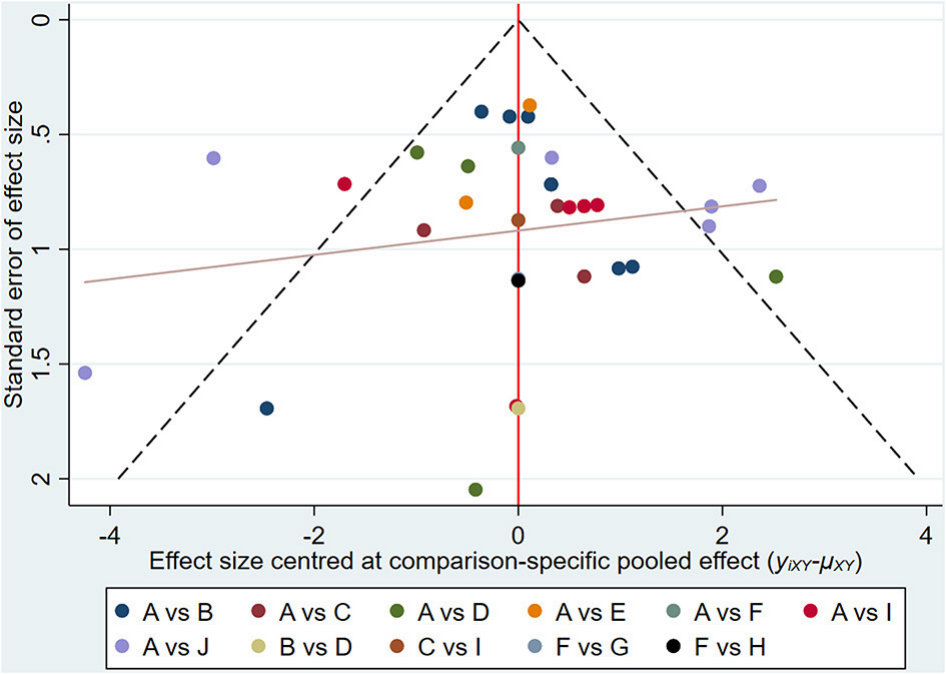
Funnel diagram for effective rate. A, TAC; B, TAC+5-FU; C, TAC+BTA; D, 5-FU; E, BLM; F, silicone; G, TAC+silicone; H, silicone+5-FU; I, BTA; J, VER. TAC, triamcinolone acetonide; 5-FU, 5-fluorouracil; BLM, bleomycin; VER, verapamil.

Results of NMA showed that compared with TAC, only TAC combined with BTA and TAC combined with 5-FU could improve efficacy rate. The difference in the efficacy rate among these therapies was statistical significant at *p* < 0.05. Interestingly, there was no statistically significant difference among other interventions at *p* < 0.05 (Figure 4; Table 4). The funnel diagram showed good symmetry, indicating no obvious publication bias. This is illustrated in Figure 5.

**Table 4:** The network meta-analysis results.

TACBTA	3.06 (0.13, 70.50)	3.29 (0.14, 80.07)	2.05 (0.01, 499.02)	1.84 (0.02, 213.83)	3.36 (0.10, 117.57)	35.90 (0.98, 1311.39)	10.26 (0.48, 221.14)	5.04 (0.20, 127.48)	0.66 (0.01, 50.09)
1.83 (0.26, 12.99)	TACFU	1.08 (0.33, 3.51)	0.67 (0.01, 66.51)	0.60 (0.02, 23.91)	1.10 (0.17, 7.29)	11.74 (1.63, 84.33)	3.35 (1.82, 6.17)	1.65 (0.57, 4.79)	0.21 (0.01, 4.86)
1.92 (0.27, 13.71)	1.05 (0.20, 5.49)	BTA	0.62 (0.01, 66.43)	0.56 (0.01, 24.30)	1.02 (0.13, 8.01)	10.91 (1.29, 92.16)	3.12 (1.13, 8.63)	1.53 (0.38, 6.11)	0.20 (0.01, 5.02)
2.07 (0.02, 192.14)	1.13 (0.02, 85.28)	1.08 (0.01, 88.42)	SiliconeFU	0.90 (0.02, 44.47)	1.64 (0.01, 219.50)	17.52 (0.13, 2419.46)	5.01 (0.05, 477.14)	2.46 (0.02, 258.63)	0.32 (0.01, 9.38)
2.14 (0.02, 197.33)	1.17 (0.02, 87.55)	1.12 (0.01, 90.79)	1.03 (0.01, 105.42)	TACSilicone	1.83 (0.03, 105.02)	19.54 (0.33, 1165.19)	5.58 (0.15, 211.12)	2.74 (0.06, 117.24)	0.36 (0.05, 2.53)
3.44 (0.36, 33.38)	1.89 (0.32, 10.98)	1.79 (0.24, 13.40)	1.66 (0.02, 145.45)	1.61 (0.02, 139.57)	FU	10.68 (0.80, 142.74)	3.05 (0.51, 18.28)	1.50 (0.20, 11.38)	0.20 (0.01, 6.76)
4.53 (0.36, 57.06)	2.48 (0.29, 20.98)	2.36 (0.24, 23.64)	2.19 (0.02, 219.82)	2.12 (0.02, 210.98)	1.32 (0.12, 14.86)	BLM	0.29 (0.04, 1.86)	0.14 (0.02, 1.15)	0.02 (0.00, 0.66)
6.77 (1.25, 36.67)	3.71 (1.37, 10.05)	3.53 (0.94, 13.21)	3.27 (0.05, 219.23)	3.16 (0.05, 210.27)	1.97 (0.43, 8.97)	1.49 (0.23, 9.87)	TAC	0.49 (0.19, 1.27)	0.06 (0.00, 1.36)
7.41 (0.94, 58.46)	4.05 (0.86, 19.14)	3.86 (0.65, 22.83)	3.58 (0.05, 282.81)	3.46 (0.04, 271.33)	2.15 (0.31, 14.88)	1.63 (0.18, 15.20)	1.09 (0.33, 3.59)	VER	0.13 (0.01, 3.21)
12.41 (0.54, 284.50)	6.80 (0.41, 113.99)	6.47 (0.34, 123.50)	6.00 (0.23, 158.62)	5.80 (0.22, 151.79)	3.60 (0.17, 75.59)	2.74 (0.11, 70.16)	1.83 (0.13, 25.62)	1.68 (0.09, 30.26)	Silicone

*Odds ratio (OR) and 95% CIs for the pairwise comparisons of the network meta-analysis from indirect comparisons, means effective rate. OR >1 indicates that the upper left intervention is more effective than the lower right intervention. Left bottom comparisons should be read from left to right. Right bottom comparisons should be read from right to left, means adverse reaction rate. OR >1 indicates that the adverse reaction rate of the upper left intervention is higher than that of the lower right intervention*.

#### Ranking Results

**Figure 6:**
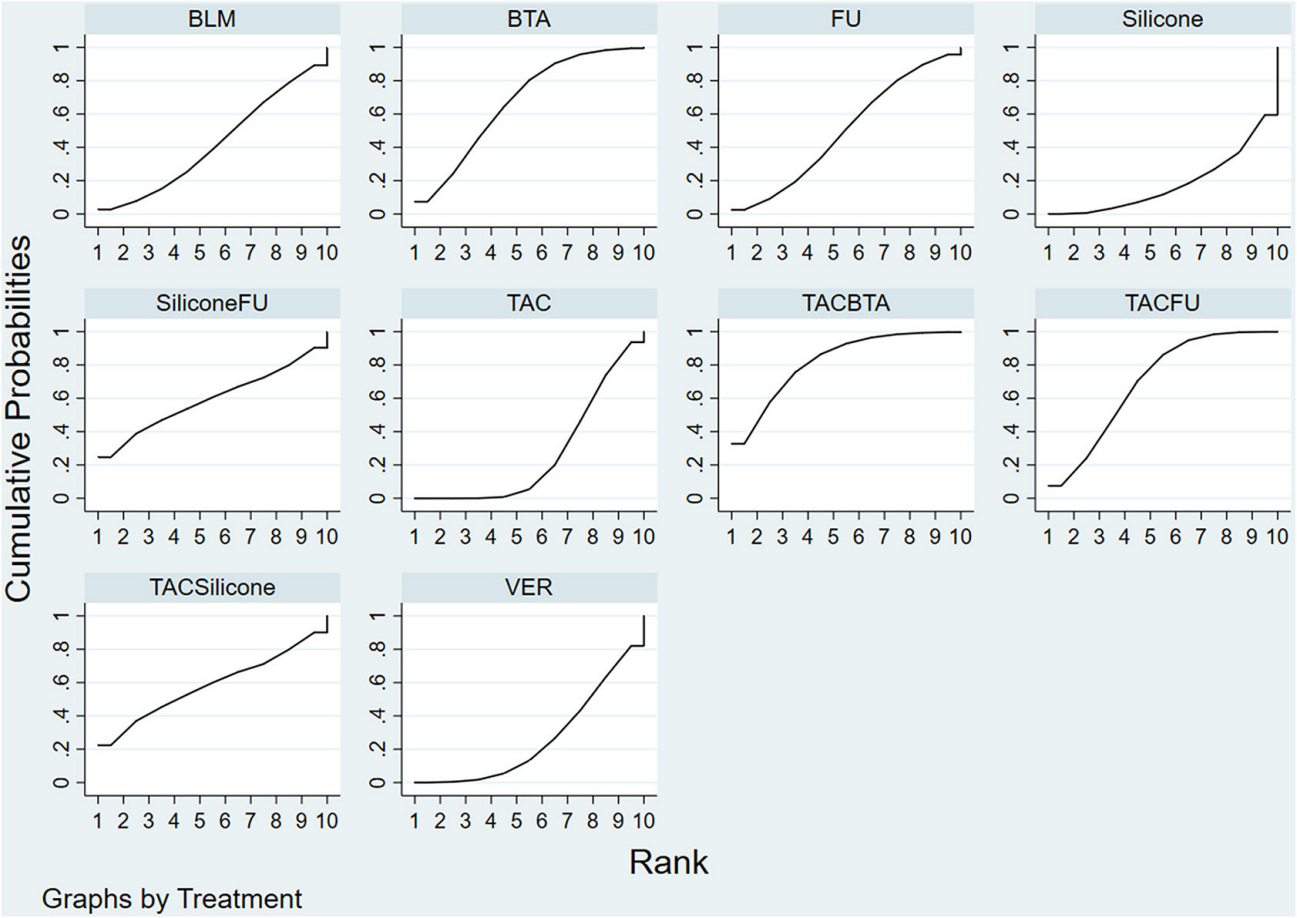
SUCRA efficacy rate ranking curve. FU, 5-FU; SiliconeFU, silicone+5-FU; TACBTA, TAC+BTA; TACFU, TAC+5-FU; TACSilicone, TAC+silicone. SUCRA, surface under the cumulative ranking; TAC, triamcinolone acetonide; 5-FU, 5-fluorouracil.

Results of the convergence analysis showed that PSRF was equal to 1, indicating that the model had good convergence. Based on the Bayesian theory of Markov chain Monte Carlo (MCMC) method, random-effects model for NMA results showed the following: TAC+BTA > TAC+5-FU > BTA > 5-FU+silicone > TAC+silicone > 5-FU > BLM > TAC > VER > silicone (Figure 6) (The dose and interval for the combination of TCA with BTA was given in Supplementary Table 1 to help readers understand the regimens easily, as this is per our study the most effective).

### Adverse Effect Rate

Adverse effect rates were reported in 21 studies (37, 40, 53–60, 62–65, 68–73, 75, 76), with a total of 10 measures. The former comprised A, TAC; B, 5-FU; C, BLM; D, Silicone gels; E, BTA, and F, VER. The latter comprised A+B, TAC combined with 5-FU; D+A, Silicone gels combined with TAC; D+B, Silicone gels combined with 5-FU; and A+E, TAC combined with BTA. The 10 treatment measures formed 45 different pair comparisons. In terms of treatment effectiveness, there are 11 direct comparisons among 21 studies; the rest were indirect comparisons. See Supplementary Figure 1.

#### Detection of Inconsistency

Eleven direct comparisons constituted one triangular closed loop. The Z-test results showed that the lower limits of 95% CI were 0. This implies that each closed loop was consistent, as shown in Supplementary Table 2. The results of the node-splitting model showed consistency in the direct and indirect comparison results, all at *p* > 0.05 (Supplementary Table 3).

#### Results of Network Meta-Analysis and Publication Bias

**Figure 7:**
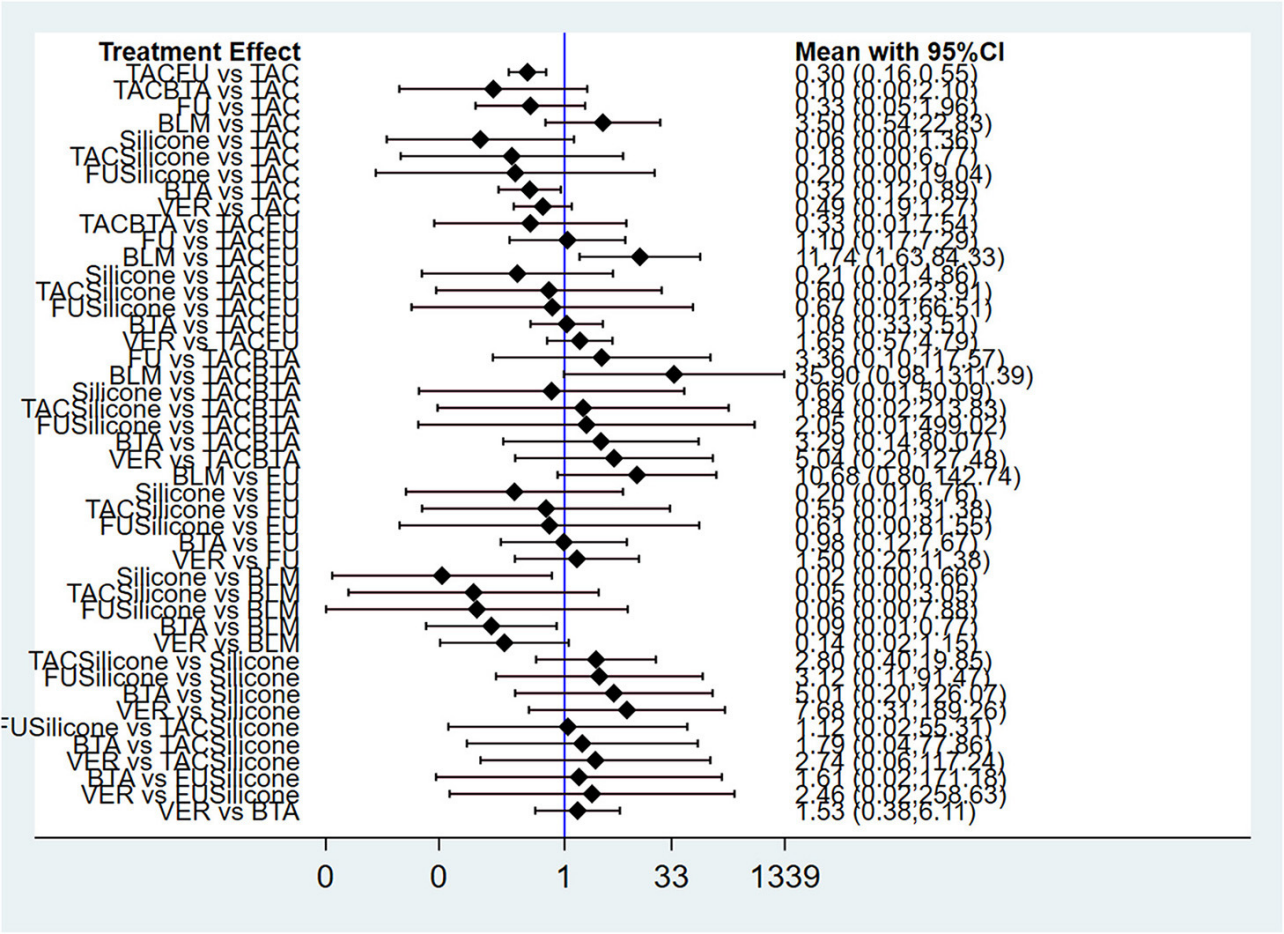
Forest plot of adverse effect rate for pairwise treatment comparison. FU, 5-FU; TACFU, TAC+5-FU; TACBTA, TAC+BTA; TACSilicone, TAC+silicone; SiliconeFU, silicone+5-FU. TAC, triamcinolone acetonide; 5-FU, 5-fluorouracil.

Results of NMA showed that compared with TAC, only TAC combined with 5-FU and BTA could decrease adverse effect rate. In addition, compared with BLM, BTA, silicone gel, and TAC combined with 5-FU could decrease adverse effect rate. The difference in the efficacy rate among these therapies was statistical significant at *p* < 0.05. Interestingly, there was no statistically significant difference among other interventions at *p* < 0.05 (Figure 7; Table 4). The funnel diagram showed good symmetry, indicating no obvious publication bias. This is illustrated in Supplementary Figure 2.

#### Ranking Results

**Figure 8:**
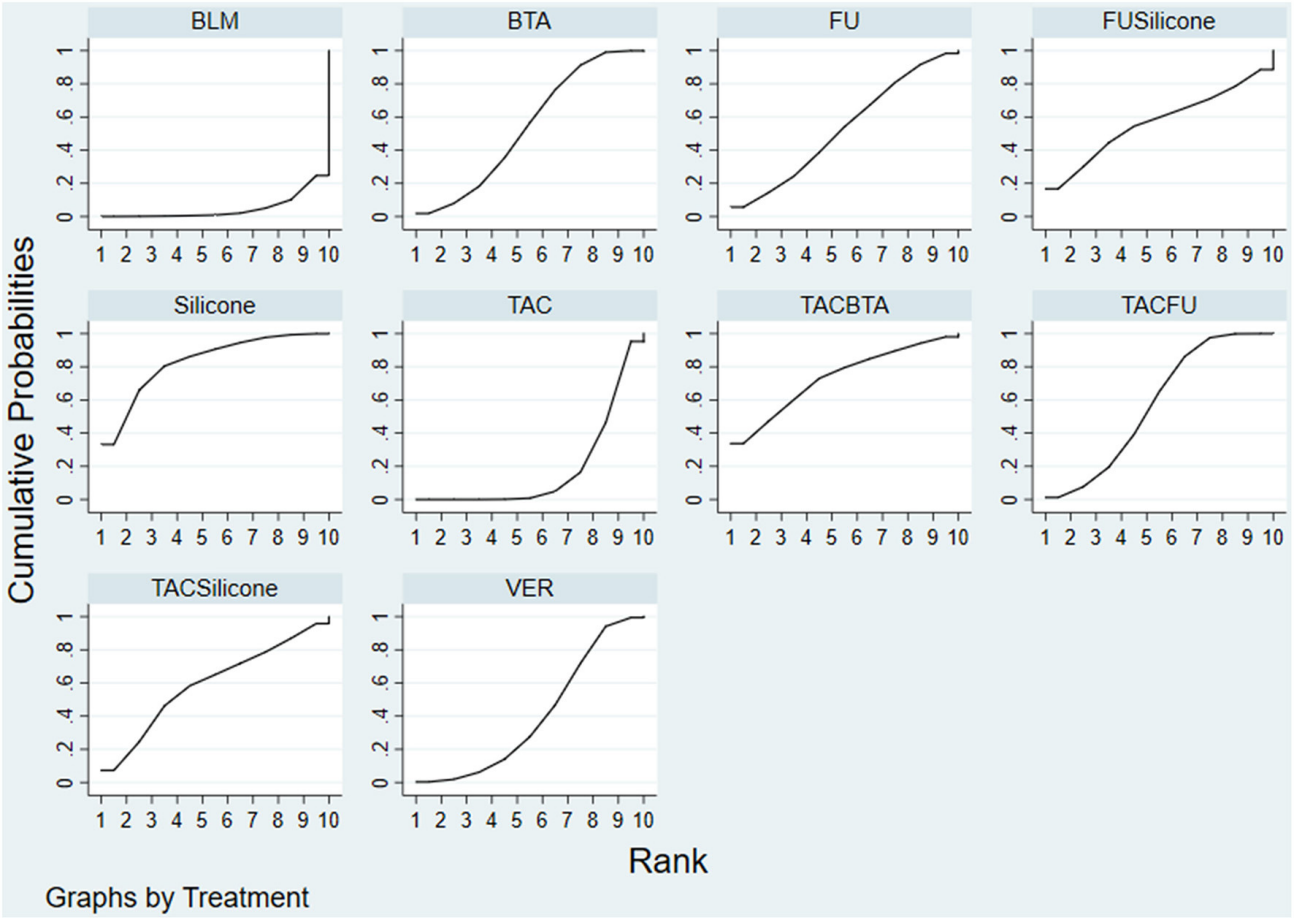
SUCRA adverse effect rate ranking curve. FU, 5-FU; SiliconeFU, silicone+5-FU; TACBTA, TAC+BTA; TACFU, TAC+5-FU; TACSilicone, TAC+silicone. SUCRA, surface under the cumulative ranking; TAC, triamcinolone acetonide; 5-FU, 5-fluorouracil.

Results of the convergence analysis showed that PSRF was equal to 1, indicating that the model had good convergence. Based on the Bayesian theory of MCMC method, use random-effects model for NMA results showed the following: silicone (83.1%) > TAC+BTA (73.4%) > TAC+silicone (59.4%) > TAC+5-FU (57.4%) > silicone+5-FU (56.5%) > BTA (54.0%) > 5-FU (52.8%) > VER (40.2%) > TAC (18.2%) > BLM (4.9%) (Figure 8).

### Recurrence Rate

Recurrence rates were reported in 11 studies (37, 40, 53–60, 62–65, 68–73, 75, 76), with a total of eight measures. The former comprised A, TAC; D, silicone gels; E, BTA; and F, VER. The latter comprised A+B, TAC combined with 5-FU; D+A, silicone gels combined with TAC; D+B, silicone gels combined with 5-FU; and A+E, TAC combined with BTA. The eight treatment measures formed 28 different pair comparisons. In terms of recurrence rate, there are eight direct comparisons among 11 studies; the rest were indirect comparisons (Supplementary Figure 3).

#### Detection of Inconsistency

Eight direct comparisons constituted one triangular closed loop. The Z-test results showed that the lower limits of 95% CI were 0. This implies that each closed loop was consistent, as shown in Supplementary Table 4. The results of the node-splitting model showed consistency in the direct and indirect comparison results, all at *p* > 0.05 (Supplementary Table 5).

#### Results of Network Meta-Analysis and Publication Bias

Results of NMA showed that compared with silicone, only TAC, VER, BTA, and BTA combined with 5-FU significantly could decrease recurrence rate. The difference in the efficacy rate among these therapies was statistical significant at *p* < 0.05. Interestingly, there was no statistically significant difference among other interventions at *p* < 0.05 (Supplementary Figure 5). The funnel diagram showed good symmetry, indicating no obvious publication bias. This is illustrated in Supplementary Figure 4.

#### Ranking Results

Results of the convergence analysis showed that PSRF was equal to 1, indicating that the model had good convergence. Based on the Bayesian theory of MCMC method, random-effects model for NMA results showed the following: VER (78%) > VER (72.7%) > BTA (70.4%) > TAC+5-FU (58.2%) > silicone+5-FU (45%) > TAC (42.4%) > silicone+TAC (29%) > silicone (4.3%) (Supplementary Figure 6).

### Efficacy and Tolerability

**Figure 9:**
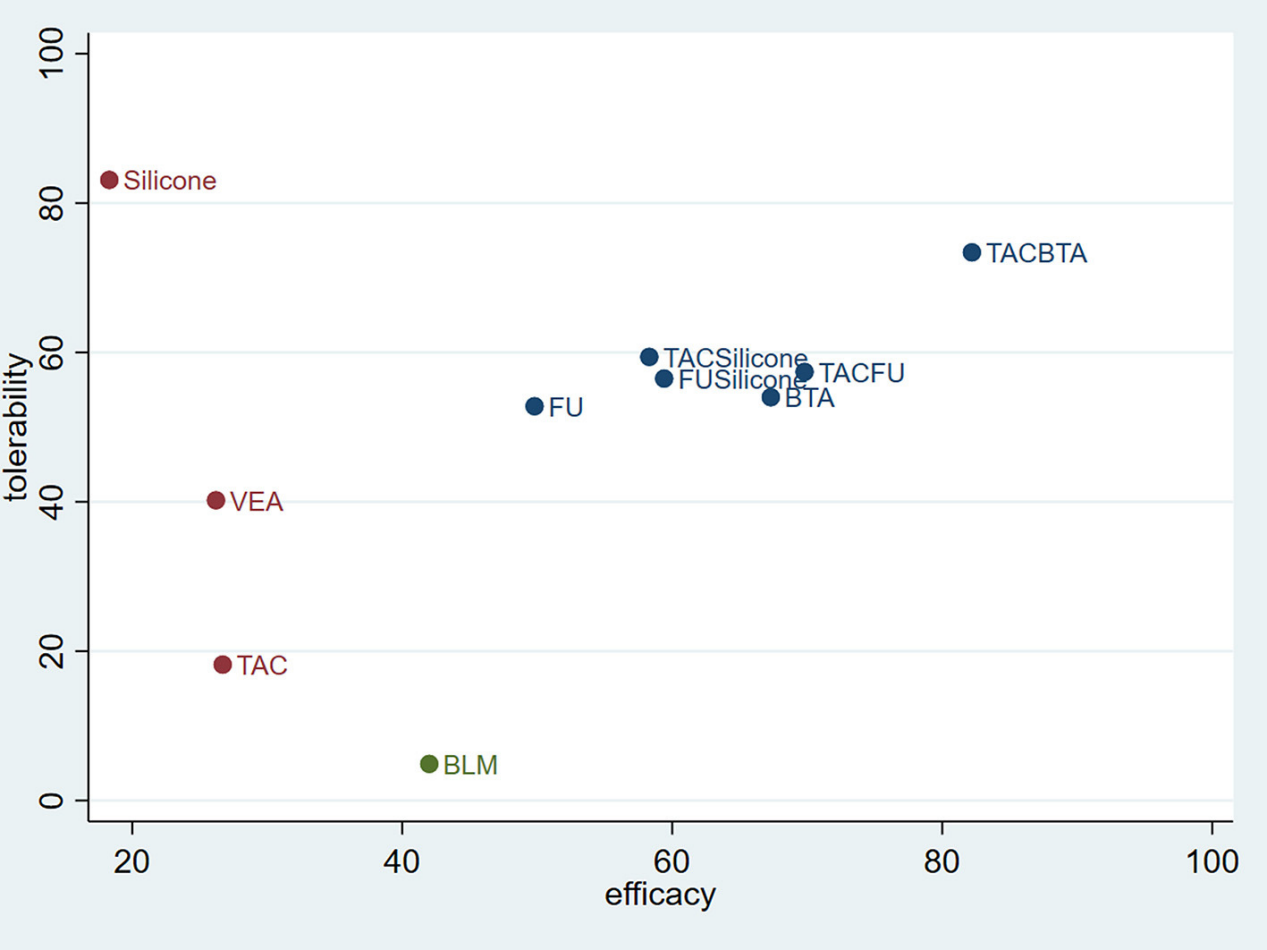
Coordinate figure for efficacy and tolerability. FU, 5-FU; SiliconeFU, silicone+5-FU; TACBTA, TAC+BTA; TACFU, TAC+5-FU; TACSilicone, TAC+silicone. TAC, triamcinolone acetonide; 5-FU, 5-fluorouracil.

From the comprehensive data, TAC combined with BTA was highly effective and well-tolerated. Furthermore, the combination therapy was superior to TAC or BTA monotherapy in terms of efficacy and tolerability. The combined therapies for BTA or TAC with 5-FU, silicone gel with TAC, and silicone gel with 5-FU revealed a better efficacy and fewer side effects. These therapies were superior to TAC, 5-FU, or silicone gel monotherapies in terms of efficacy and tolerability. Silicone gels had the best tolerance but poor therapeutic response. On the other hand, BLM had poor tolerance (Figure 9).

## Discussion

At present, pathological scars are still an unsolved problem worldwide. The readily available drugs and methods for treating pathological scars are limited mainly due to incomplete understanding of the mechanism of scar formation. Comparative analysis on the methods for treating pathological scars like hypertrophic scar and keloid is critical for clinical management (43). Traditional meta-analysis does not compare and analyze the effects of three or more treatments. Therefore, in the present study, a network meta-analysis was performed to evaluate the efficacy and safety of injection with TAC, 5-FU, BTA, VER, BLM, and commonly used topical drugs in the treatment of hypertrophic scar and keloid.

The results of NMA showed that the combined therapies were better than the two therapeutic modalities separately. Good efficacy and tolerability were revealed when TAC was combined with BTA, followed by TAC combined with 5-FU. These findings imply that TAC-BTA and TAC-5-FU therapies are more effective and safer in the treatment of keloid and hypertrophic scars. Silicone gels may be considered good for patients who cannot tolerate side effects. A previous meta-analysis by Ren et al. concluded that TAC+5-FU was safer and more effective in the treatment of pathological scars than TAC. Also, the effect of combined medication was significantly better than that of 5-FU (77). In recent years, a meta-analysis also found that TAC combined with 5-FU in the treatment of keloid and hypertrophic scars was more effective and safer than the monotherapy of TAC and 5-FU (78). The authors further reported that TAC combined with 5-FU was more effective in the treatment of keloid and hypertrophic scars than was intralesional injection TAC. The combined therapy showed significant improvements in scar height, erythema, and Observer and Patient Scar Assessment Scale. Most studies support that combined therapy is safer and more acceptable to patients, with fewer complications and a lower recurrence rate than intralesional TAC. In the present study, the effective concentration of TAC was 10 to 40 mg/ml for monotherapy, but how many units of BTA and TAC were used in combination therapy was an underexamined area. However, on administering a combined therapy of 5-FU and TAC, the concentration of TAC was far lower than the effective concentration. Therefore, we hypothesized that TAC might play an anti-inflammatory role in the combination therapy and offset most of the side effects of 5-FU, but further research is needed to verify this.

BTA inhibits muscle fiber contraction, reduces the tension at the wound healing edge (33), and enhances retention of fibroblasts in the GO and G1 phases of the cell cycle (34). Meanwhile, decreased expression of TGF-β1 may directly regulate fibroblast activity by changing apoptosis migration and fibrosis, thereby reducing scar formation (35, 36). Similar to our findings, a meta-analysis that included high-quality studies showed that intralesional injection of BTA was more effective in treating pathological scars than TAC or placebo (79). Also, it significantly reduced post-injection pain (79). A recent meta-analysis evaluating the efficacy and safety of BLM, TAC, 5-FU, TAC combined with 5-FU, and TAC combined with cryotherapy found that BLM significantly improved the treatment of pathological scars than TAC or 5-FU combined with cryotherapy (80). There was no significant difference between the BLM group and the TAC combined with 5-Fu group. In addition, their results showed that BLM reduced the recurrence rate when compared with 5-FU-TAC combined therapy. These results suggest that BLM is a more effective treatment option for keloid or hypertrophic scars than TAC and 5-FU or their combined therapies. However, the adverse reaction rate of BLM was higher (80). Interestingly, these findings are not in line with the current study's findings. This may be because the recurrence rate after BLM treatment was not reported in our included RCTs. Also, this study included controlled clinical trials, and the outcome indicators for our evaluation of the efficacy were different.

In the present study, it was revealed that BTA, glucocorticoid combined with 5-FU, VER, and 5-FU showed no statistically significant difference in the therapeutic effect. The efficacy of glucocorticoid combined with 5-FU was better than that of glucocorticoid and VER. The difference was, however, not statistically significant compared with 5-FU. Elsewhere, an NMA involving 23 studies with a total of 1,513 patients found that BTA combined with glucocorticoids had the best effect on pathological scar (45). Unlike the current study, which only included TAC, their study included four injectable drugs, including TAC and Diprospan. Another NMA sought to evaluate the efficacy of TAC in the treatment of keloids compared with placebo, pulsed dye laser (PDL), 5-FU, silicone, VER, TAC+5-FU, and TAC+5-FU+PDL (46). The authors suggested that VER is preferable to other treatments. Different from our study, their study only compared the treatment of keloid, did not include hypertrophic scar, and only included 10 RCTs.

## Limitations

However, there are also limitations in our study. First of all, the included trials were followed up for a short time, ranging from 12 weeks to 24 months. The duration of the follow-up for 23 subjects was <32 weeks. Previous studies have found that 50–94% of pathological scars recurred after 1 year of treatment (81, 82). Nonetheless, long-term follow-up can be difficult because most patients lose interest in follow-up after achieving satisfactory treatment results. Secondly, hypertrophic scar and keloid were reported simultaneously in several of the studies we included (38, 40, 74, 83). Although they are both fibroproliferative diseases, they should not be confused with each other (84, 85), which may be an important cause of heterogeneity. Thirdly, the dose of each treatment and the interval between each treatment varied. Fourthly, all the studies were conducted in selected countries and thus cannot be used to generalize conclusions of the study. Scarring is a worldwide disease, and it is valuable to know how different regions and different races respond to the same treatment. Fifthly, none of the included studies classified patients according to Fitzpatrick's skin type. It is currently believed that Fitzpatrick's skin type is an important factor affecting the occurrence and development of pathological scars. Therefore, this study does not reveal whether there is a difference in the therapeutic effect of different treatment measures for different Fitzpatrick's skin types. Sixthly, although no publication bias was found according to the funnel plot, potential bias risks still exist, such as the lack of covert random allocation methods and financial support from the pharmaceutical industry, which may lead to overestimation of efficacy. Seventhly, our study did not assess the efficacy of physical or surgical modalities such as cryotherapy, lasers, and excision. Eighthly, the diagnosis of the scars in some included studies was clinical only and not confirmed by histopathology. Finally, it is important to note that adding botulinum toxin that is not covered by public insurance may have had a limit to its use.

## Conclusion

Based on the Bayesian theorem, the consistency model was selected to conduct an NMA to evaluate the relative efficacy and safety of TAC, 5-FU, BLM, BTA, and VER in treatment of pathological scars. According to the main data synthesis, we concluded that more combination therapies should be recommended for the treatment of pathological scars, especially the TAC-5-FU and TAC-BTA combined therapies. These therapies have the advantage of good curative effects and fewer side effects. But for patients who cannot tolerate the side effects, we recommend the use of silicone gels in combination with TAC. However, there are still shortcomings in this NMA, and its conclusions need to be further confirmed by well-designed and rigorous RCTs.

## Data Availability Statement

The original contributions presented in the study are included in the article/Supplementary Material, further inquiries can be directed to the corresponding author/s.

## Author Contributions

SY designed the study, processed the data, conducted statistical analyses, interpreted data, drafted article, approved the final version, and is the guarantor. CL contributed to study design, interpreted data, drafted article, and approved the final version. YL contributed to interpretation of data, drafted article, and approved the final version. All authors contributed to the article and approved the submitted version.

## Conflict of Interest

The authors declare that the research was conducted in the absence of any commercial or financial relationships that could be construed as a potential conflict of interest.

## Publisher's Note

All claims expressed in this article are solely those of the authors and do not necessarily represent those of their affiliated organizations, or those of the publisher, the editors and the reviewers. Any product that may be evaluated in this article, or claim that may be made by its manufacturer, is not guaranteed or endorsed by the publisher.
