# Supplementary material for: Network Meta-Analysis of Different Clinical Commonly Used Drugs for the Treatment of Hypertrophic Scar and Keloid
Source: Front Med (Lausanne). 2021 Sep 9;8:691628. doi: 10.3389/fmed.2021.691628 (PMC8458741; doi:10.3389/fmed.2021.691628)
Supplement: Supplementary file 1 [file Data_Sheet_1.doc]

Supplementary Table 1. The dose and interval for the combination of TCA with BTA.

| First author, year | Dose and interval |
| --- | --- |
| Hend D. Gamil,  2019 | patients subjected to intralesional combined therapy of BTA followed by TAC in the same lesion. BTA injections were done 0.1 ml until clinically visible slight blanching. The dosage was adjusted to 2.5 IU cm^3^ of the lesion with a maximum dosage of 100 I session. The TAC injections were done at a dose of 0.1-cm^3^ of involved skin with a maximum dosage of the session. Injections were done monthly for a total of three sessions. |
| Peng Cheng, 2015 | patients subjected to intralesional combined therapy of BTA followed by TAC in the same lesion. 1h after surface anesthesia ointment, BTA injections were done 1 IU/point at a spacing of 1cm, using multipoint injection techniques. Two weeks after, inject TAC, the dosage was 2 mg /cm^2^ of the lesion with a maximum dosage of 50 mg/session. Injections were done every two weeks for a total of five sessions. |
| Zhenzhen Li, 2016 | patients subjected to intralesional combined therapy of BTA followed by TAC in the same lesion. 1h after surface anesthesia ointment, BTA injections were done 1 IU/point at a spacing of 1cm, using multipoint injection techniques. Two weeks after, inject TAC, the dosage was 2 mg /cm^2^ of the lesion with a maximum dosage of 50 mg/session. Injections were done every two weeks for a total of five sessions. |

Supplementary Table 1. Inconsistency detection of closed loop for adverse effects rate.

| Loop | p value | 95% CI |
| --- | --- | --- |
| TAC--TAC+TBA--TBA | 0.537 | (0.00, 6.42) |

Supplementary Table 3. Results of node splitting model for adverse effects rate.

| Side | A C | A F | C I | F G | F H |
| --- | --- | --- | --- | --- | --- |
| p value | 0.324 | 0.980 | 0.324 | 0.983 | 0.990 |

A: TAC, C: TAC+BTA, F: Silicone, G:TAC+Silicone, H: Silicone+5-FU, I: BTA.

Supplementary Table 4. Inconsistency detection of closed loop for recurrence rate.

| Loop | p value | 95% CI |
| --- | --- | --- |
| TAC--TAC+TBA--TBA | 0.170 | (0.00, 6.84) |

Supplementary Table 5. Results of node splitting model for recurrence rate.

| Side | A C | A D | C G | D E | D F |
| --- | --- | --- | --- | --- | --- |
| p value | 0.684 | 0.975 | 0.684 | 0.979 | 0.986 |

A: TAC, C: TAC+BTA, D: 5-FU, E: BLM, F: Silicone, G:TAC+Silicone.
