# Supplementary material for: Network Meta-Analysis of Different Clinical Commonly Used Drugs for the Treatment of Hypertrophic Scar and Keloid
Source: Front Med (Lausanne). 2021 Sep 9;8:691628. doi: 10.3389/fmed.2021.691628 (PMC8458741; doi:10.3389/fmed.2021.691628)
Supplement: Supplementary file 2 [file Table_2.DOC]

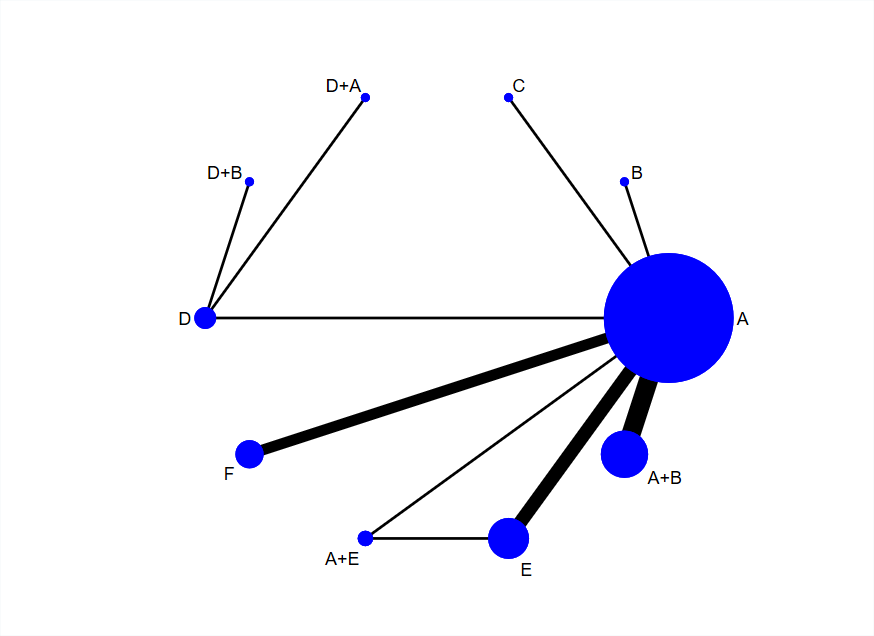


Supplementary Figure 1. Network diagram of adverse effects rate. A: TAC, B: 5-FU, C: BLM, D: Silicone, E: BTA, F:VER, A+B: TAC + 5-FU, D+A: Silicone + TAC, D+B: Silicone + 5-FU, A+E: TAC + BTA


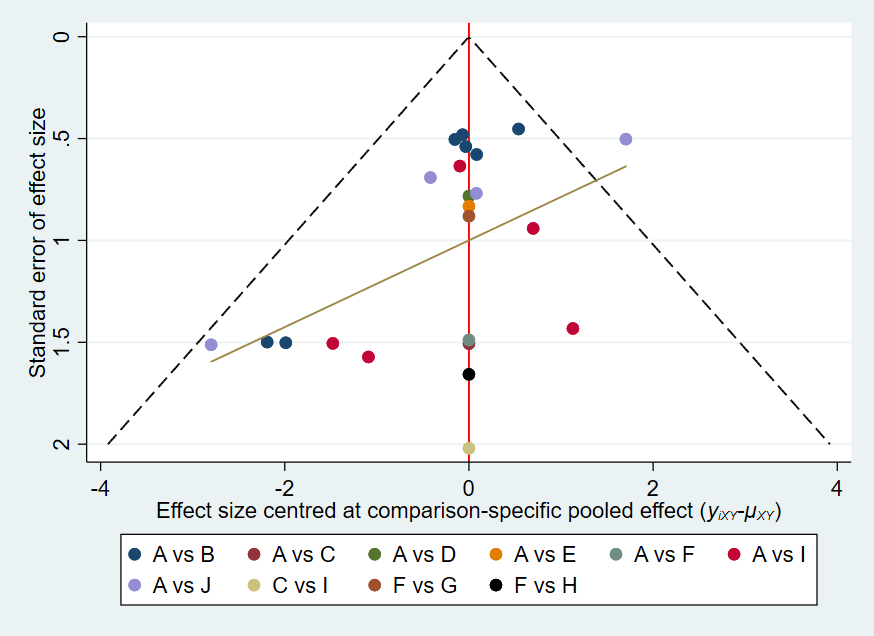


Supplementary Figure 2. Funnel diagram for adverse effects rate. A: TAC, B: TAC+5-FU, C: TAC+BTA, D: 5-FU, E: BLM, F: Silicone, G:TAC+Silicone, H: Silicone+5-FU, I: BTA, J:VER.


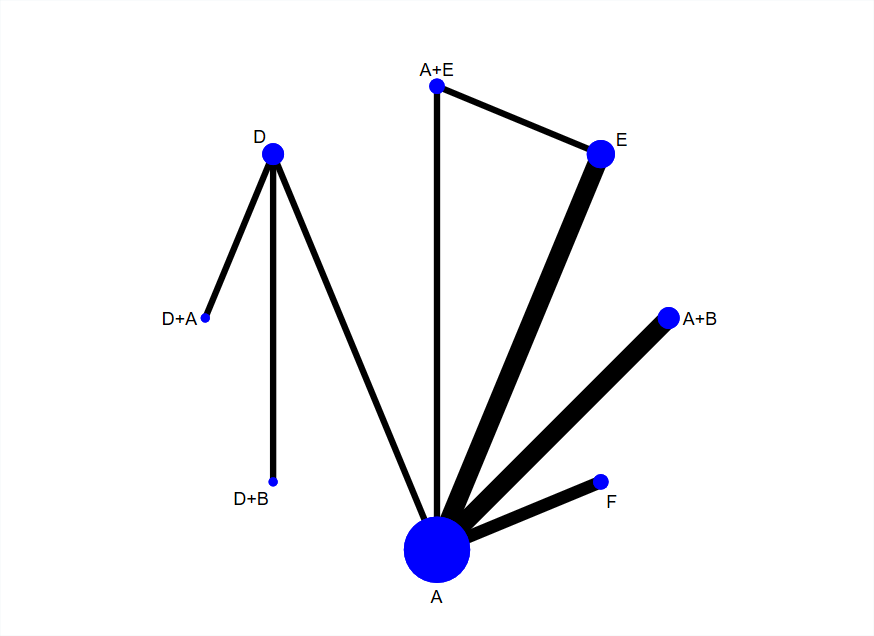


Supplementary Figure 3. Network diagram of recurrence rate. A: TAC, B: 5-FU, C: BLM, D: Silicone, E: BTA, F:VER, A+B: TAC + 5-FU, D+A: Silicone + TAC, D+B: Silicone + 5-FU, A+E: TAC + BTA


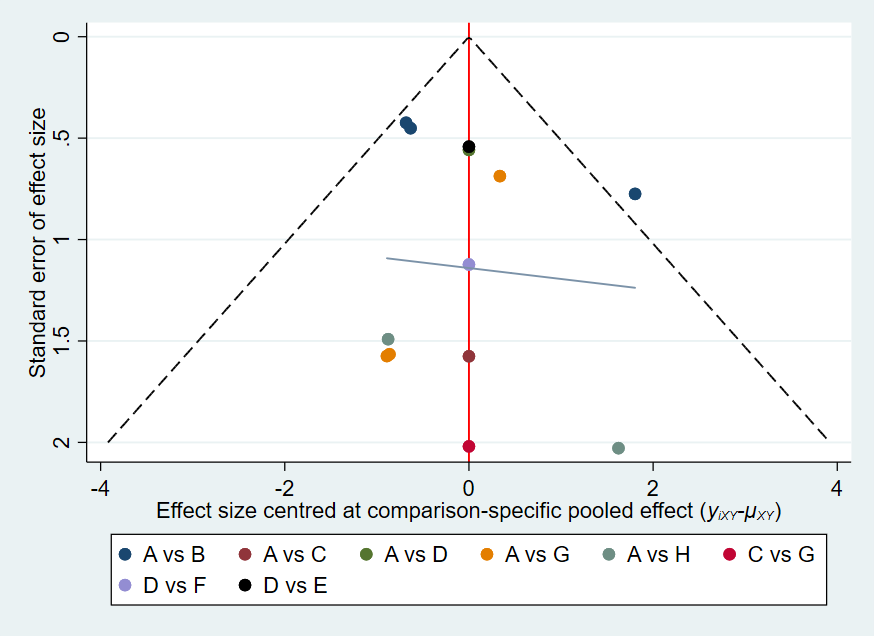


Supplementary Figure 4. Funnel diagram for recurrence rate. A: TAC, B: TAC+5-FU, C: TAC+BTA, D: 5-FU, E: BLM, F: Silicone, G:TAC+Silicone, H: Silicone+5-FU, I: BTA, J:VER.


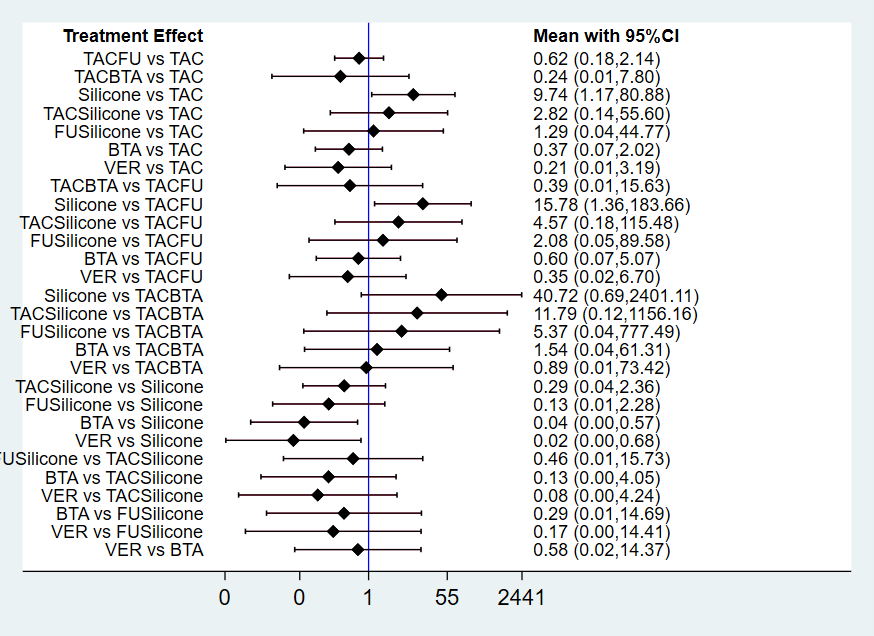


Supplementary Figure 5. Forest plot of recurrence rate for pairwise treatment comparison. FU：5-FU, TACFU: TAC+5-FU, TACBTA: TAC+BTA, TACSilicone: TAC+Silicone, SiliconeFU: Silicone+5-FU.


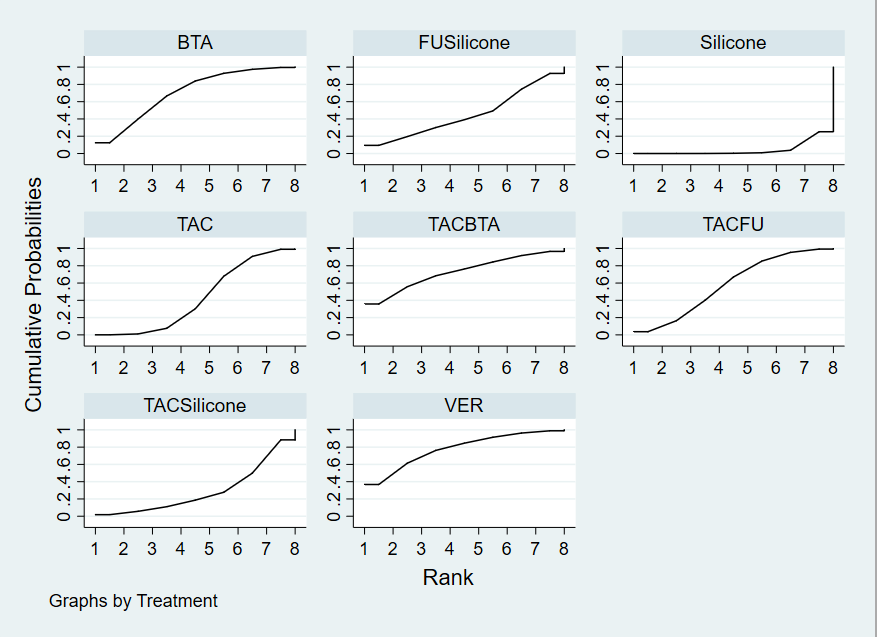


Supplementary Figure 6. SUCRA recurrence rate ranking curve. FU：5-FU, SiliconeFU: Silicone+5-FU, TACBTA: TAC+BTA, TACFU: TAC+5-FU, TACSilicone: TAC+Silicone.
